# Metabolomic Signatures of Healthy Dietary Patterns Associated With Lower Risk of Age-Related Macular Degeneration

**DOI:** 10.1167/iovs.67.13.11

**Published:** 2026-11-04

**Authors:** Haemin Kang, Huan Yun, Shuhua Xu, Jun Li, Kevin Mendez, Ines Lains, Archana Nigalye, Raviv Katz, Krupa Sourirajan, Roshni Bhat, Georgiy Kozak, Hanna Choi, Augustine Bannerman, Rodrigo A. Alvarez, David Wu, Ivana K. Kim, John B. Miller, Demetrios G. Vavvas, Joan W. Miller, Jessica Lasky-Su, Liming Liang, Deeba Husain

**Affiliations:** 1 Massachusetts Eye and Ear, Department of Ophthalmology, Harvard Medical School, Boston, Massachusetts, United States; 2 Department of Epidemiology, Harvard T. H. Chan School of Public Health, Boston, Massachusetts, United States; 3 Department of Nutrition, Harvard T. H. Chan School of Public Health, Boston, Massachusetts, United States; 4 Division of Preventive Medicine, Department of Medicine, Brigham and Women's Hospital and Harvard Medical School, Boston, Massachusetts, United States; 5 Channing Division of Network Medicine, Department of Medicine, Brigham and Women's Hospital and Harvard Medical School, Boston, Massachusetts, United States; 6 Department of Biostatistics, Harvard T. H. Chan School of Public Health, Boston, Massachusetts, United States

**Keywords:** age-related macular degeneration (AMD), diet, metabolomics, optical coherence tomography (OCT)

## Abstract

**Purpose:**

The purpose of this study was to evaluate the association of metabolomic signatures reflecting adherence and metabolic response to dietary patterns with prevalence and structural severity of age-related macular degeneration (AMD).

**Methods:**

In this cross-sectional study of 1008 eyes from 504 participants, metabolomic profiles in plasma and urine were measured by liquid chromatography-tandem mass spectrometry. Previously derived signatures for eight dietary patterns—including the Alternate Mediterranean diet (AMED), Dietary Approaches to Stop Hypertension (DASH), plant-based diet indices (PDIs), and two mechanism-based diets including Empirical Dietary Inflammatory Pattern (EDIP) and Empirical Dietary Index for Hyperinsulinemia (EDIH)—were evaluated for association with AMD using linear mixed models and Mendelian randomization (MR).

**Results:**

Each signature (38–66 metabolites) correlated with its dietary pattern score (Pearson *r* = 0.16–0.30). Per SD increase in plasma signatures of four healthy dietary patterns (AMED, DASH, PDI, and healthy PDI [hPDI]) were associated with 12% to 16% lower odds of AMD and reduced severity, whereas the unhealthy PDI (uPDI) signature was associated with 13% higher odds and greater severity (false discovery rate [FDR] < 0.05). DASH showed the strongest inverse association with AMD and with atrophy, hyper-reflective foci, and ellipsoid-zone disruption (β = −0.808 to −0.613). Further MR analyses found that genetically instrumented signatures of DASH and Alternate Health Eating Index (AHEI)-2010 were inversely associated with AMD, whereas the EDIP signature was positively associated.

**Conclusions:**

Metabolomic signatures of healthy dietary patterns are associated with lower odds and reduced structural severity of AMD. Further longitudinal studies are warranted to validate these findings.

Age-related macular degeneration (AMD) is the leading cause of blindness in adults over the age of 50 years, and the number of affected individuals is expected to double by 2050.^1^^–^^3^ Early prevention through healthy lifestyle modifications, such as smoking, diet, and physical activity, is promising, with diet receiving increasing attention.^4^ Dietary patterns, which capture the synergistic effects of various foods, nutrients, and beverages, provide a useful framework for evaluating the impact of overall diet on health.^5^ Accumulating evidence suggests that adherence to a Mediterranean diet—characterized by higher intake of vegetables, whole grains, and nuts—is associated with a reduced risk of progression from early to late AMD.^6^^,^^7^ Plant-based diets are rich in antioxidants, which may reduce oxidative stress and help prevent dry AMD.^8^ In contrast, higher dietary inflammatory potential^9^ and consumption of high-glycemic index foods^10^ have been linked to an increased risk of AMD. Their comparability in relation to AMD remains underexplored, limiting translation into public dietary recommendations. Moreover, the mechanisms through which dietary patterns influence AMD are not yet fully elucidated.

Metabolomics, the comprehensive measurement of intermediate and end products of dietary intake, gut microbiota activity, and endogenous metabolism, holds promise in assessing metabolic responses to dietary exposures and their biological effects.^11^ Emerging studies have identified numerous individual metabolites associated with specific foods^12^ and composite dietary patterns, such as the Mediterranean diet^13^ and the Dietary Approaches to Stop Hypertension (DASH).^14^ However, most studies have focused on individual metabolites and often fail to account for the synergistic effects among diet-related metabolites, thereby limiting their ability to evaluate the overall metabolic impact of dietary patterns. More recently, multi-metabolite scores—termed “metabolomic signatures”—have been proposed to capture metabolic responses to dietary intake and their complex interactions with host factors, including genetics and the gut microbiome, thereby providing a more objective complement to traditional dietary assessments.^15^ For instance, Li et al.^16^ derived a metabolomic signature comprising 67 metabolites for the Mediterranean diet and found that this signature could predict future cardiovascular disease risk independent of known risk factors. Despite this progress, whether metabolomic signatures of various dietary patterns are associated with AMD risk remains unclear. Addressing this gap is important for advancing our understanding of the relationship between diet and AMD, as well as the underlying biological mechanisms.

To test this hypothesis, in a population of older adults at high risk of AMD, we leveraged metabolomic and clinical data to examine the associations of metabolomic signatures—measuring adherence to and metabolic responses to dietary patterns—with AMD risk, as well as retinal structural characteristics assessed by optical coherence tomography (OCT). In secondary analyses, we further evaluated whether genetic variants associated with these metabolomic signatures were also related to AMD risk.

## Methods

### Study Population

This cross-sectional study is a part of a prospective observational study at Massachusetts Eye and Ear, Harvard Medical School, Boston, Massachusetts.^17^^–^^21^ Since January 2015, we have consecutively recruited participants from the Retina Service as well as the Comprehensive Ophthalmology and Optometry Services at Massachusetts Eye and Ear Infirmary during their regular appointments. We included patients over the age of 50 years with the diagnosis of AMD and a control arm with no AMD. Exclusion criteria included diagnosis of any other vitreoretinal disease, active uveitis or ocular infection, significant media opacities that precluded the observation of the ocular fundus, refractive error of 6 diopters or more of spherical equivalent, history of retinal surgery, history of any ocular surgery or intraocular procedure, such as laser and intraocular injections within the 90 days before enrollment, and diagnosis of diabetes mellitus, with or without concomitant diabetic retinopathy. A standardized medical history questionnaire was administered to collect data on smoking habits, current information of weight and height for body mass index (BMI), and systemic diseases.^17^^–^^21^ Smokers were defined as those who reported current smoking, and former smokers as those who have ever smoked, regardless of when they stopped, but who do not currently smoke. All participants underwent a comprehensive eye examination, including non-stereoscopic seven-field color fundus photography and spectral-domain OCT imaging.

In the present study, we included participants with available diet and plasma metabolome data, resulting in 504 individuals (1008 eyes) for the main analysis. Further, we excluded those without urinary metabolome data within this set, leaving 200 participants (400 eyes) in secondary analysis. The participant flowchart is depicted in Supplementary Figure S1. The research protocol was conducted in accordance with Health Insurance Portability and Accountability Act requirements and the tenets of the Declaration of Helsinki and was approved by the Institutional Review Board of Massachusetts Eye and Ear Infirmary (2019P002725). All included participants provided written informed consent.

### Dietary Assessment

Diet was assessed using a validated semi-quantitative food frequency questionnaire (FFQ) developed by the Department of Nutrition, Harvard T. H. Chan School of Public Health, Boston, Massachusetts, USA. The reproducibility of the FFQ has been validated elsewhere.^22^ For the current analysis, we calculated eight well-studied dietary pattern scores: three guideline-recommended diets, three plant-based diets, and two mechanism-driven diets reflecting dietary inflammatory or hyperinsulinemic potential. Each score was computed from the FFQ using previously published scoring algorithms^23^^–^^29^; component definitions and scoring are provided in the Supplementary Methods.

### Metabolomic Profiling in Both Plasma and Urine

Fasting samples of plasma and urine were meticulously gathered and preserved at −80°C, as previously described.^17^^–^^21^ Following stringent protocols for shipping and storage, these samples were carefully packaged on dry ice and dispatched to Metabolon, Inc. in Morrisville, North Carolina, USA, for metabolomic analysis. At Metabolon, Inc., plasma specimens underwent non-targeted mass spectrometry utilizing ultra-high-performance liquid chromatography-tandem MS (UPLC-MS/MS). This involved using four distinct ionization techniques: “PosEarly” and “PosLate” for hydrophilic and hydrophobic compounds, respectively, utilizing a Waters UPLC BEH C18 column; “Neg” using basic negative ion conditions on a C18 column; and “Polar” with negative ionization on a Waters UPLC BEH Amide column. Each method was accompanied by MS and MSn scans. The analysis of urine samples followed a similar UPLC-MS/MS methodology, using a Waters ACQUITY ultra-UPLC and a Thermo Scientific Q-Exactive spectrometer interfaced with a heated electrospray ionization (HESI-II) source and Orbitrap mass analyzer. This comprehensive approach ensured precise analysis of both plasma and urine samples, enabling the capture of a broad spectrum of metabolites under diverse conditions. Data were then normalized at Metabolon, Inc. via batch normalization. For urine samples, the normalized data were further adjusted to osmolality to accommodate concentration differences. After quality control, a total of 897 metabolites in plasma and 453 in urine were measured. For metabolite data, missing values were imputed using half of minimum values given the low concentration below detection.

### Plasma and Urinary Metabolomic Signatures of Dietary Patterns

We previously developed metabolomic signatures for eight dietary patterns using interdisciplinary elastic net modeling in two lifestyle validation studies with detailed dietary phenotyping.^30^ These signatures were discovered and validated among populations with diverse age, sex, race/ethnicity, yielding mean Pearson correlations of 0.39 in the training set and 0.32 in the test sets, and were then applied to the current study population. We calculated eight metabolomic scores using the established formula: metabolomic signature = intercept + β_1_x_1_ + β_2_x_2_ + … + β_n_x_n_. The metabolites were standardized using z-score transformation before calculation. To explore whether the urine metabolomic profile could serve as a surrogate approach, we then applied the same formula to calculate the corresponding metabolomic signatures using urine metabolome data. The number of available metabolites in plasma and urine used to derive these signatures is shown in Supplementary Table S1. Over 60% metabolites were available in the AMD cohort, enough for applying metabolomic signatures as shown in previous studies.^16^

### Ascertainment of Age-Related Macular Degeneration

Color fundus photographs were used for ascertaining AMD. The AMD staging was based on color fundus photography by design, because the Age-Related Eye Disease Study (AREDS) 2 severity scale applied here was developed and validated on color fundus photographs, and because our prior studies in this cohort used the same photograph-based grading, ensuring comparability across analyses. OCT images were not used for staging; they were graded separately for structural phenotypes, as described below. Two independent experienced graders analyzed all field 2-color fundus photographs on IMAGEnet 2000 software (version 2.56; Topcon Medical Systems).^31^ In cases of disagreement, a senior author established the final categorization. All graders were masked to the patients’ clinical and demographic characteristics during this process. AMD was staged according to the AREDS 2 severity scale as control (AREDS stage 1; drusen maximum size smaller than circle C0 and total area less than C1), early AMD (stage 2; drusen maximum size C0 or larger but smaller than C1, or AMD-characteristic pigment abnormalities), intermediate AMD (stage 3; drusen maximum size C1 or larger, or drusen of C0 or larger exceeding the specified total area), and late AMD (stage 4; presence of geographic atrophy or evidence of exudative AMD). Geographic atrophy was defined as a lesion 433 µm or larger in diameter (AREDS circle I-2) showing at least 2 of the following: absence of retinal pigment epithelium pigment, circular shape, or sharp margins, irrespective of foveal involvement. Staging was based on color fundus photography alone. Late AMD (referred to as advanced AMD in Table 1) comprised eyes with geographic atrophy, eyes with evidence of exudative AMD, and eyes with both; these were analyzed together as a single stage rather than as separate atrophic and exudative subtypes; full grading definitions are provided in the Supplementary Methods.^32^

**Table 1.** Baseline Characteristics of the Study Population (1008 Eyes of 504 Participants)

Characteristic	Healthy, 276 Eyes	Early AMD, 88 Eyes	Intermediate AMD, 401 Eyes	Advanced AMD, 243 Eyes	*P*_overall_
Age, y	68.3 (7.95)	70.4 (7.43)	72.8 (7.72)	78.5 (8.08)	<0.001
Male, *n*	104 (37.7%)	30 (34.1%)	152 (37.9%)	82 (33.7%)	0.67
Race/ethnicity, *n*					0.21
Non-Hispanic	260 (94.2%)	78 (88.6%)	377 (94.0%)	211 (86.8%)	
Hispanic	6 (2.17%)	5 (5.68%)	5 (1.25%)	8 (3.29%)	
Others	10 (3.62%)	5 (5.68%)	19 (4.74%)	24 (9.88%)	
Smoking status, *n*					0.002
Never	157 (57.3%)	54 (61.4%)	189 (47.1%)	82 (34.0%)	
Current smoker	8 (2.92%)	0 (0.00%)	7 (1.75%)	7 (2.90%)	
Previous smoker	109 (39.8%)	34 (38.6%)	205 (51.1%)	152 (63.1%)	
BMI, kg/m^2^	27.3 (5.62)	26.3 (4.37)	27.3 (5.48)	27.6 (6.18)	0.32
Dietary pattern score					
AMED	5 (4, 6)	6 (4, 7)	5 (3, 6)	5 (3, 6)	0.25
AHEI-2010	63 (55, 70)	62 (54, 72)	59 (50, 69)	58 (51, 64)	0.067
DASH	24 (20, 27)	24 (21, 27)	23 (21, 27)	23 (20, 26)	0.96
PDI	53 (48, 55)	52 (49, 57)	52 (49, 56)	53 (48, 56)	0.82
hPDI	48 (44, 52)	48 (44, 51)	48 (43, 51)	47 (43, 51)	0.30
uPDI	49 (45, 53)	49 (46, 53)	49 (44, 54)	49 (44, 54)	0.69
EDIP	−0.07 (−0.19 to 0.09)	−0.14 (−0.29 to 0.09)	−0.03 (−0.25 to 0.13)	−0.05 (−0.18 to 0.10)	0.52
EDIH	0.30 (0.15, 0.46)	0.27 (0.11, 0.47)	0.28 (0.15, 0.49)	0.33 (0.15, 0.50)	0.88
Plasma metabolomic signature					
AMED	4.65 (4.20, 4.98)	4.57 (4.22, 4.93)	4.57 (4.15, 5.03)	4.52 (4.05, 4.93)	0.89
AHEI-2010	68.6 (66.4, 72.3)	70.0 (68.2, 72.0)	68.8 (65.7, 72.2)	68.3 (64.4, 71.4)	0.16
DASH	24.4 (23.4, 25.3)	24.6 (23.6, 25.2)	24.1 (23.0, 25.3)	23.7 (22.4, 24.8)	0.003
PDI	53.2 (52.6, 54.2)	53.3 (52.7, 54.2)	53.1 (52.3, 54.1)	53.2 (52.4, 54.0)	0.30
hPDI	54.6 (53.4, 56.0)	54.5 (53.4, 55.7)	54.2 (52.8, 55.9)	54.0 (52.5, 55.6)	0.45
uPDI	53.7 (52.2, 55.0)	53.4 (52.4, 55.3)	54.0 (52.6, 55.1)	54.0 (52.7, 55.5)	0.29
EDIP	−0.11 (−0.19 to −0.05)	−0.15 (−0.19 to −0.07)	−0.14 (−0.23 to −0.06)	−0.12 (−0.19 to −0.04)	0.19
EDIH	0.38 (0.33, 0.44)	0.38 (0.35, 0.43)	0.39 (0.33, 0.44)	0.40 (0.34, 0.45)	0.52

AMD, age-related macular degeneration; AMED, alternative Mediterranean diet; AHEI-2010, alternate healthy eating index 2010; BMI, body mass index; DASH, dietary approaches to stop hypertension; EDIH, empirical dietary index for hyperinsulinemia; EDIP, empirical dietary inflammatory pattern; hPDI, healthy plant-based diet index; PDI, plant-based diet index; SD, standard deviation; uPDI, unhealthy plant-based diet index.

Data is presented as mean (SD) or median (interquartile range) for continuous variables and *n* (percentage) for categorical variables.

### Optical Coherence Tomography Features of Age-Related Macular Degeneration

Two independent graders, masked to any clinical characteristics, assessed baseline OCT images for the presence of classic drusen, subretinal drusenoid deposits (SDDs), ellipsoid zone (EZ) disruption, hyper-reflective foci, drusenoid pigmented epithelial detachment (PED), serous PED, chorioretinal atrophy, intraretinal fluid (IRF) and subretinal fluid (SRF) ADDIN EN.CITE.^10^ Presence of intraretinal and/or subretinal fluid on OCT was recorded as evidence of exudative AMD. Because fluorescein angiography was not performed, these OCT findings indicate exudation rather than angiographically confirmed neovascularization. In any case of disagreement, the third grader resolved the disagreement. After OCT grading, we also computed a composite sum score representing the number of AMD-related structural features present in each eye by summing eight individual OCT features (range = 0–8).^19^

### Statistical Analysis

Baseline participant characteristics are depicted as mean with standard deviation (SD) or median with interquartile range (IQR) for continuous variables and n (percentage) for categorical variables. Pearson correlation coefficients (*r*) were calculated among conventional dietary scores and corresponding metabolomic signatures in both plasma and urine.

Presence of AMD (no AMD or AMD) and severity of AMD (no AMD, early AMD, intermediate AMD, and late AMD) were set as the dependent variables for the statistical analysis. AMD severity was graded according to the AREDS 2 criteria, with late AMD (AREDS stage 4) defined as the presence of geographic atrophy or evidence of exudative AMD; full grading definitions are provided in the Supplementary Methods. Because this study is cross-sectional, all reported associations reflect prevalent AMD; the term “risk” is used to denote associations with prevalent disease rather than incident disease. To account for two eyes’ data from the same subject, multilevel mixed regression models were used to estimate associations of dietary scores and metabolomic signatures with AMD severity (no AMD, early, intermediate, and late AMD) as well as the sum scores of OCT features. Before analysis, both dietary scores and metabolomic signatures were inverse-normal transformed to render a normal distribution. In model 1, we adjusted for age and sex. In model 2, we further adjusted for race, smoking, and BMI. We also simultaneously included dietary scores and metabolomic signatures of corresponding dietary patterns to examine the association independence. Generalized linear mixed model was used to estimate associations of dietary scores and metabolomic signatures with risk of AMD and eight OCT features. For binary outcomes (AMD presence and each individual OCT feature), these models were fitted on the logit scale, so the reported β coefficients represent the change in log-odds per SD increment of the dietary score or metabolomic signature, with the corresponding odds ratio (OR) obtained as exp(β). Same covariates were adjusted in the models.

The association of individual metabolites constituting the metabolomic signatures with food components of each dietary pattern and AMD severity were then analyzed using generalized linear regression models. In sensitivity analyses, we analyzed the associations in the non-Hispanic subpopulation. In addition, we stratified the population by age, sex, smoking status, and BMI, respectively, and repeated the association analyses.

A two-sample Mendelian randomization (MR) analysis was further performed to investigate the putative causal effect between dietary metabolomic signatures and risk of AMD. We obtained summary data for AMD from the International AMD Genomics Consortium (16,144 late-stage AMD cases and 17,832 AMD-free controls),^33^ whereas the summary data for dietary metabolomic signatures was obtained from the recently published data (*n* = 13,700).^30^ For each metabolomic signature, independent genome-wide significant variants were used as genetic instruments, yielding two to nine instruments per signature (40 single-nucleotide polymorphisms [SNPs] in total). The F-statistic exceeded 10 for every instrument (range = 11 to 433), indicating no evidence of weak-instrument bias. The overall instrumental estimate of the effect of any given exposure on outcome was calculated using the penalized robust inverse variance weighting (IVW) method as the primary analysis, the weighted median test, mode-based estimation (MBE), MR-Egger regression, and leave-one-out validation were conducted as sensitivity analyses.

All statistical tests were two-sided. To account for multiple comparisons, the Benjamini–Hochberg procedure was applied to control the false discovery rate (FDR) at <5%. All analyses were conducted using R software (version 4.2.3).

## Results

### Characteristics of the Study Population

A total of 1008 eyes from 504 participants with complete dietary and plasma metabolomics data were included in the main analysis (see Table 1). The mean baseline age was 72.7 ± 8.7 years, and 184 participants (36.5%) were men. Of the 504 participants, 463 (91.9%) were non-Hispanic, 12 (2.4%) were Hispanic, and 29 (5.8%) did not know or declined to report their ethnicity. Race was recorded separately but is not presented in Table 1 owing to the very small numbers in non-White categories; among the non-Hispanic participants, all but two self-identified as White (one was Asian and one was Black). Compared with the control group (68.3 ± 8.0 years), participants in AMD groups were likely to be older (70.4 ± 7.4 years in early AMD, 72.8 ± 7.7 years in intermediate AMD, and 78.5 ± 8.1 years in advanced AMD; *P* < 0.001) and former smokers (*P* = 0.002). Characteristics of the subpopulation with additional urinary metabolomics data are presented in Supplementary Table S2 and showed a similar pattern to the overall cohort.

### Adherence to Dietary Patterns: Dietary Scores and Metabolomic Signatures

The metabolomic signatures, each comprising 38 to 66 metabolites from plasma or urine, showed good comparability with the corresponding FFQ-derived dietary scores but exhibited narrower variation (Supplementary Fig. S2).

Dietary pattern scores showed moderate to high correlations within the same categories: 0.63 to 0.68 among the 3 healthy diets, –0.63 to 0.34 among the 3 plant-based diets, and 0.64 between the 2 mechanism-driven diets. Plasma metabolomic signatures were significantly correlated with their respective dietary scores (Fig. 1; all *P* < 0.001). Similarly, urinary metabolomic signatures were strongly correlated with plasma signatures (Pearson *r* = 0.58–0.71, all *P* < 0.001) and also showed significant associations with dietary scores (all *P* < 0.017).

**Figure 1.**
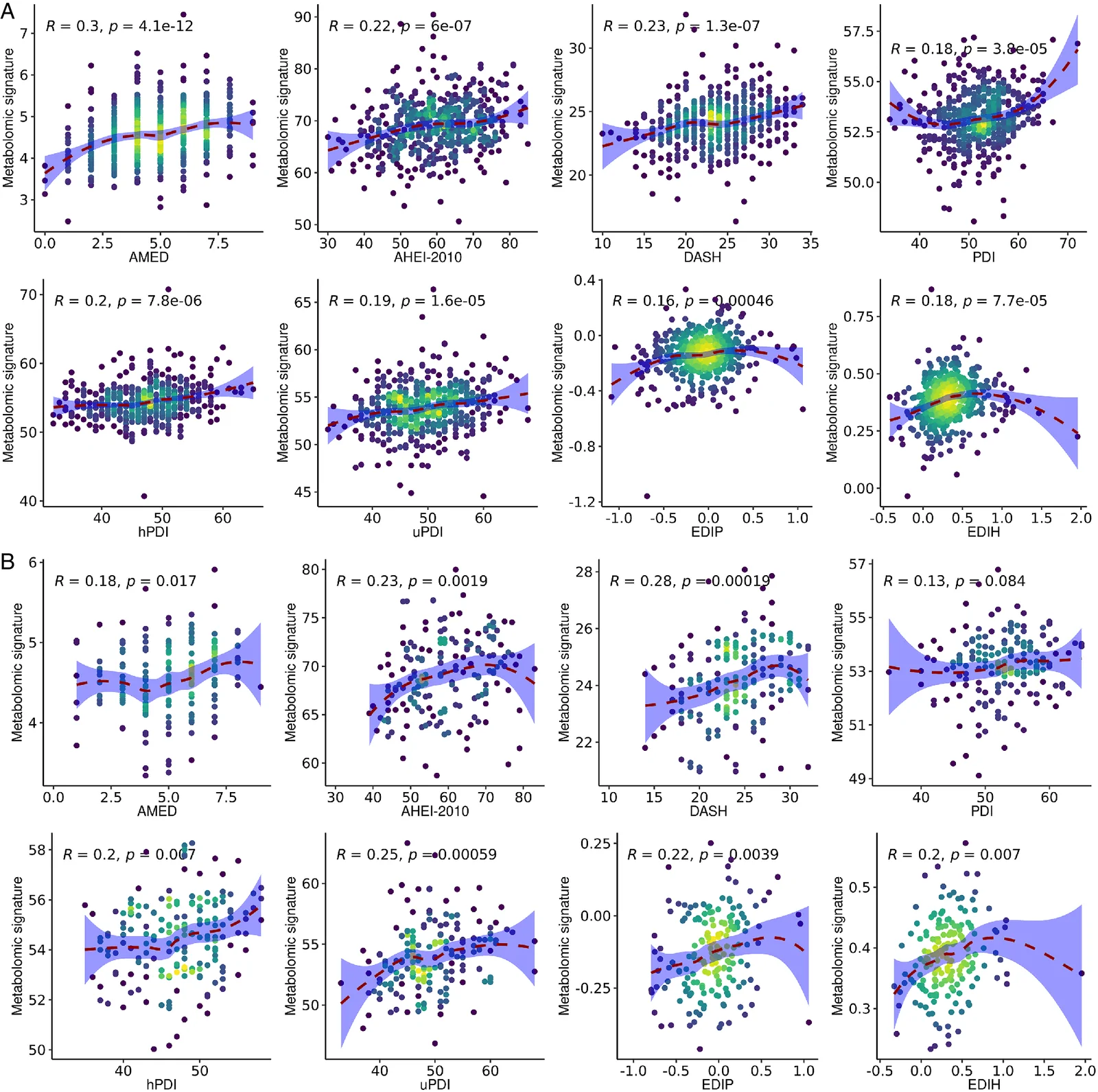
**Pearson correlation between dietary pattern scores and corresponding metabolomic signatures in plasma (A) and urine (B).** AMED, alternative Mediterranean diet; AHEI-2010, alternate healthy eating index 2010; DASH, dietary approaches to stop hypertension; EDIH, empirical dietary index for hyperinsulinemia; EDIP, empirical dietary inflammatory pattern; hPDI, healthy plant-based diet index; PDI, plant-based diet index.

### Association of Dietary Metabolomic Signatures With Age-Related Macular Degeneration Risk

After multivariable adjustment for age, sex, race, smoking, and BMI, five plasma metabolomic signatures were significantly associated with presence of AMD: Alternate Mediterranean diet (AMED; OR = 0.88, 95% confidence interval [CI] = 0.80–0.98, *P* = 0.020), DASH (OR = 0.84, 95% CI = 0.76–0.94, *P* = 0.002), plant-based diet index (PDI; OR = 0.86, 95% CI = 0.77–0.95, *P* = 0.004), healthy PDI (hPDI; OR = 0.86, 95% CI = 0.77–0.96, *P* = 0.006), and unhealthy PDI (uPDI; OR = 1.13, 95% CI = 1.02–1.25, *P* = 0.016), whereas no significant associations were observed for the corresponding dietary scores (Table 2). Among these five signatures, only the urinary metabolomic signature for PDI was associated with AMD risk (OR = 0.85, 95% CI = 0.73–1.00, *P* = 0.048; Supplementary Table S3).

**Table 2.** Association of Dietary Scores and Plasma Metabolomic Signatures With Risk of Age-Related Macular Degeneration^†^

	Model 1^‡^		Model 2^§^	
	OR (95% CI)	*P* Value	OR (95% CI)	*P* Value
Dietary pattern score				
AMED	0.94 (0.86, 1.03)	0.21	0.96 (0.86, 1.07)	0.46
AHEI-2010	0.91 (0.83, 0.99)	0.037	0.92 (0.83, 1.03)	0.14
DASH	0.96 (0.88, 1.05)	0.38	0.99 (0.89, 1.09)	0.81
PDI	0.97 (0.88, 1.05)	0.43	0.97 (0.88, 1.08)	0.61
hPDI	0.94 (0.86, 1.02)	0.14	0.94 (0.85, 1.05)	0.28
uPDI	0.98 (0.90, 1.07)	0.65	0.97 (0.87, 1.07)	0.53
EDIP	1.03 (0.94, 1.13)	0.49	1.00 (0.90, 1.10)	0.97
EDIH	1.05 (0.96, 1.15)	0.29	1.00 (0.91, 1.11)	0.92
Metabolomic signature				
AMED	0.88 (0.81, 0.96)	0.005^*^	0.88 (0.80, 0.98)	0.020^*^
AHEI-2010	0.91 (0.83, 0.99)	0.03^*^	0.93 (0.83, 1.03)	0.18
DASH	0.85 (0.78, 0.93)	<0.001^*^	0.84 (0.76, 0.94)	0.002^*^
PDI	0.88 (0.80, 0.96)	0.003^*^	0.86 (0.77, 0.95)	0.004^*^
hPDI	0.86 (0.79, 0.94)	0.001^*^	0.86 (0.77, 0.96)	0.006^*^
uPDI	1.13 (1.04, 1.24)	0.006^*^	1.13 (1.02, 1.25)	0.016^*^
EDIP	0.98 (0.90, 1.07)	0.67	0.94 (0.85, 1.04)	0.26
EDIH	1.09 (1.00, 1.19)	0.048	1.05 (0.95, 1.17)	0.35

* FDR-corrected *P* value < 0.05.

† Odds ratio (OR) and 95% confidence interval (CI) of AMD presence per standard deviation increment in dietary pattern score or metabolomic signature using generalized linear mixed model.

‡ Model 1: adjusted for age, sex.

§ Model 2: further adjusted for race, smoking, and BMI.

Consistent with the results using AMD as a binary variable, these plasma metabolomic signatures were also significantly associated with AMD severity. These associations remained significant after further adjustment for the corresponding dietary scores (Fig. 2). In a sensitivity analysis restricted to the non-Hispanic subpopulation, the associations between metabolomic signatures and AMD severity were largely unchanged (Supplementary Fig. S3). Stratified analyses by age, sex, smoking status, and BMI suggested that these associations were more pronounced among older participants, men, and former smokers. No significant heterogeneity was observed in subgroup analyses (*P*_interaction_ > 0.05; Supplementary Table S4). Similarly, among urinary metabolomic signatures, only the PDI signature was significantly associated with AMD severity (β = –0.157, *P* = 0.048; Supplementary Table S5).

**Figure 2.**
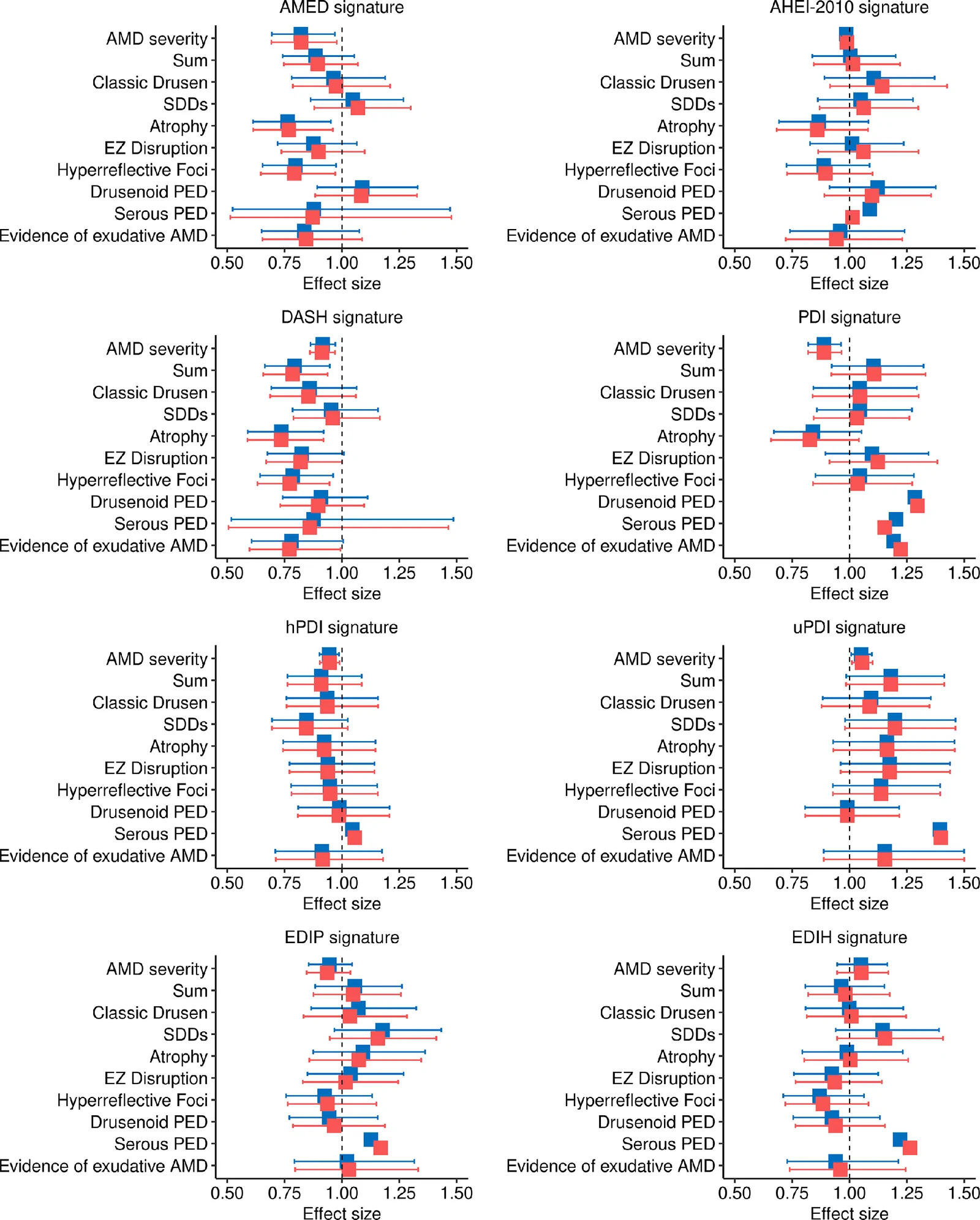
**Association of dietary metabolomic signatures with AMD severity and OCT features.** Data are shown as the beta and 95% confidence interval per standard deviation increment in dietary metabolomic signatures, adjusted for age, sex, race, smoking, and BMI. AMD, age-related macular degeneration; AMED, alternative Mediterranean diet; AHEI-2010, alternate healthy eating index 2010; BMI, body mass index; DASH, dietary approaches to stop hypertension; EDIH, empirical dietary index for hyperinsulinemia; EDIP, empirical dietary inflammatory pattern; EZ, ellipsoid zone; hPDI, healthy plant-based diet index; PDI, plant-based diet index; PED, pigment epithelial detachment; SDD, subretinal drusenoid deposit; uPDI, unhealthy plant-based diet index.

### Association of Dietary Metabolomic Signatures With Optical Coherence Tomography Features

As OCT imaging features allow for more refined subclassification of AMD phenotypes,^10^ we further examined whether the dietary metabolomic signatures were associated with specific OCT features. In multivariable-adjusted models, three AMD-related metabolomic signatures, including AMED, DASH, and PDI, showed significant associations with one or more OCT features (FDR < 0.05; see Fig. 2). Among these, the DASH signature demonstrated the strongest inverse associations, being linked to atrophy (β = −0.613, *P* = 0.011), hyper-reflective foci (β = −0.808, *P* = 0.002), EZ disruption (β = −0.773, *P* = 0.029), and the composite OCT feature score (β = −0.190, *P* = 0.017). Similar patterns were observed in the non-Hispanic subpopulation (see Supplementary Fig. S3). Although the associations of the DASH signature with these OCT features appeared more pronounced among individuals younger than 65 years, men, and those with obesity, no statistically significant interactions were detected (Supplementary Fig. S4).

In secondary analyses, approximately 6 to 11 individual metabolites within the plasma metabolomic signatures were significantly associated with AMD severity after multiple testing correction (Supplementary Figs. S5–S12). Notably, sphingomyelins, such as SM(d18:1/20:0) and SM(d18:1/18:0), along with ceramide d18:1/16:0, showed the strongest positive associations (effect sizes = 0.21–0.26), highlighting a potential role of the sphingomyelin pathway in AMD pathogenesis. In contrast, asparagine, betaine, and C10 carnitine were inversely associated with AMD severity. Several plasma metabolites associated with AMD were also related to specific OCT features such as atrophy, EZ disruption, and classic drusen. For example, SM(d18:1/24:1) was significantly associated with EZ disruption (β = 0.804, *P* = 0.019), classic drusen (β = 1.328, *P* = 0.002), and the composite OCT feature score (β = 0.267, *P* = 0.001). Furthermore, SM(d18:1/18:0) and SM(d18:1/20:0) were inversely associated with the monounsaturated fatty acid/saturated fatty acid (MUFA/SFA) ratio and vegetable oil intake (see Supplementary Fig. S5).

### Mendelian Randomization Analysis Between Dietary Metabolomic Signatures and Age-Related Macular Degeneration

We further performed a two-sample MR analysis to examine whether genetically instrumented dietary metabolomic signatures were associated with AMD. Detailed information of included variants is listed in Supplementary Table S6. In the primary IVW analysis, the DASH (OR = 0.88. 95% CI = 0.78–0.99, *P* = 0.038) and Alternate Health Eating Index (AHEI)-2010 (OR = 0.84, 95% CI = 0.74–0.95, *P* = 0.005) signatures were inversely associated with AMD, whereas the Empirical Dietary Inflammatory Pattern (EDIP) signature was positively associated (OR = 1.73, 95% CI = 1.12–2.68, *P* = 0.013). The AMED and PDI signatures were not significant under IVW (*P* = 0.88 and *P* = 0.062, respectively) but were inversely associated in the pleiotropy-robust weighted median and mode-based estimators, which we interpret as exploratory given the small number of genetic instruments (Table 3, Supplementary Fig. S13). No strong evidence of heterogeneity and unbalanced pleiotropy existed. However, nonsignificant associations with AMD were detected for hPDI and uPDI as we found in observational analysis. In leave-one-out sensitivity analysis, the relationship of AMED (effect size ranged from 0.83–0.88), AHEI-2010 (effect size ranged from 0.78–0.85), and EDIP (effect size ranged from 1.71–1.96) with risk of AMD were attenuated but remained still significant when excluding most of SNPs from the analysis (Supplementary Fig. S14).

**Table 3.** Mendelian Randomization Analysis Between Dietary Metabolomic Signatures and Risk of Age-Related Macular Degeneration^†^

	MR-IVW		MR-MBE		MR-WM		MR-Egger	
Trait	OR (95% CI)	*P* Value	OR (95% CI)	*P* Value	OR (95% CI)	*P* Value	OR (95% CI)	*P* Value
AMED	0.97 (0.62, 1.51)	0.88	0.81 (0.71, 0.93)	0.002*	0.83 (0.72, 0.97)	0.019*	0.85 (0.40, 1.79)	0.67
AHEI-2010	0.84 (0.74, 0.95)	0.005*	0.81 (0.70, 0.94)	0.004*	0.79 (0.69, 0.92)	0.002*	0.84 (0.69, 1.03)	0.087
DASH	0.88 (0.78, 0.99)	0.038	0.80 (0.68, 0.93)	0.004*	0.81 (0.70, 0.95)	0.011*	0.76 (0.60, 0.96)	0.022
PDI	0.79 (0.62, 1.01)	0.062	0.83 (0.69, 1.00)	0.044	0.75 (0.59, 0.94)	0.015*	0.85 (0.55, 1.31)	0.45
hPDI	0.96 (0.78, 1.19)	0.72	1.03 (0.87, 1.23)	0.70	1.05 (0.88, 1.24)	0.59	1.22 (0.83, 1.78)	0.31
uPDI	1.15 (0.97, 1.36)	0.10	1.13 (0.53, 2.41)	0.75	1.08 (0.85, 1.36)	0.54	1.06 (0.81, 1.37)	0.68
EDIP	1.73 (1.12, 2.68)	0.013*	1.80 (1.10, 2.95)	0.019*	1.97 (1.13, 3.45)	0.017*	1.12 (0.53, 2.37)	0.77
EDIH	0.91 (0.62, 1.33)	0.64	1.13 (0.74, 1.75)	0.57	1.09 (0.74, 1.60)	0.68	0.60 (0.20, 1.81)	0.36

CI, confidence interval; IVW, inverse-variance weighted; MBE, mode-based estimation; MR, Mendelian randomization; OR, odds ratio; WM, weighted median.

* FDR-corrected *P* value < 0.05.

† Summary data for AMD outcome was from International AMD Genomics Consortium (16,144 late-stage AMD cases and 17,832 AMD-free controls), whereas the summary data for metabolomic signatures was from our recently published data (*n* = 13,700).

## Discussion

In our study, metabolomic signatures of four healthy dietary patterns were associated with a lower prevalence and reduced severity of AMD. In contrast, metabolomic signature of the uPDI was associated with a higher prevalence and greater severity of AMD, independent of conventional risk factors. Among these, the DASH signature showed the strongest inverse associations with AMD risk and several OCT-derived features. Additionally, several sphingomyelins, inversely associated with the MUFA/SFA ratio and vegetable oil intake, may mediate the relationship between these healthy dietary patterns and AMD risk. These findings suggest that dietary metabolomic signatures, as a complement to traditional dietary assessments, may help identify individuals at high risk of AMD and provide deeper biological insights into the role of diet in its pathogenesis.

To our knowledge, this is the first study to systematically investigate metabolomic profiles of multiple dietary patterns in relation to AMD. Healthy dietary patterns—typically rich in vegetables, fruits, nuts, and other nutrient-dense foods—are thought to slow down AMD progression by reducing oxidative stress and inflammation.^7^^,^^34^^,^^35^ Whereas previous studies have highlighted the beneficial role of the Mediterranean diet in AMD, little attention has been given to other dietary patterns.^35^^,^^36^ In the present study, associations between healthy diets and AMD risk assessed using conventional dietary questionnaires were inverse but not statistically significant, possibly due to limited sample size or measurement error inherent in self-reported dietary assessment; importantly, because metabolomic signatures integrate dietary intake, intestinal absorption, host metabolism, and gut-microbial activity, they are expected to reflect dietary exposure more sensitively and comprehensively than self-reported scores. In a cohort of this size—smaller than the large dietary-survey cohorts in which conventional dietary scores were originally validated—this greater sensitivity is the most plausible explanation for why the metabolomic signatures, but not the questionnaire-based scores, reached statistical significance. Moreover, these signatures were derived to predict diet in independent cohorts rather than fitted to AMD in the present sample, which makes it unlikely that their associations simply reflect disease-related metabolic changes. Nevertheless, reverse causation cannot be excluded. Circulating metabolites are dynamic and may themselves be altered by AMD-related processes, including oxidative stress, chronic inflammation, and lipid dysregulation, or by disease-associated changes in appetite and dietary behavior. Because exposure and outcome were measured at the same time point, the present design cannot establish the direction of these associations, and the signatures may partly reflect AMD-related metabolic alterations rather than upstream dietary exposure alone. For example, Shah et al. identified metabolite signatures for multiple food groups and dietary indices in a cohort of 2259 adults using sparse canonical correlation analysis.^37^ Our findings extend this concept to AMD and suggest that diet-related metabolomic profiles may provide valuable insights beyond conventional dietary assessments.

Beyond evaluating AMD risk and staging using color fundus photography, we further investigated the relationship between dietary metabolomic signatures and retinal structural features using OCT. The OCT imaging of the retina has become the preferred modality for AMD evaluation and monitoring and provides detailed insights into disease manifestations.^38^ In this study, plasma metabolomic signatures including AMED, DASH, and uPDI were associated with OCT features of AMD, including hyper-reflective foci and retinal layer integrity such as atrophy and EZ disruption. Hyper-reflective foci are thought to represent migration of retinal pigment epithelial cells toward the retinal surface and have been proposed as structural endpoints for studies aiming to prevent progression from intermediate to advanced AMD.^39^ EZ disruption reflects photoreceptor damage and may serve as a surrogate marker of intermediate AMD.^40^ The observed inverse associations with healthy dietary signatures suggest that these diets may help preserve retinal structural integrity and slow progression of dry AMD, potentially through metabolic regulation. Metabolite-level analyses highlighted several pathways that may underlie the observed diet–AMD associations, including sphingomyelin, ceramide, and lysophosphatidylcholine (LPC) metabolism. Of them, SM(d18:1/18:0), SM(d18:1/20:0), and SM(d18:1/24:1) showed strong positive associations with AMD severity and several OCT features. For example, SM(d18:1/24:1) was associated with EZ disruption, classic drusen, and the composite OCT score, whereas Cer (d18:1/16:0) were associated with atrophy. Sphingomyelin and ceramide are sphingolipids containing a sphingosine backbone and are involved in apoptosis, autophagy, inflammation, and cellular stress responses.^41^^–^^43^ Experimental studies suggest that dysregulated sphingolipid metabolism may contribute to AMD development, as demonstrated in acid sphingomyelinase knockout mice.^43^ Ceramide metabolism in photoreceptors also appears to be altered under oxidative stress conditions.^44^ LPC is a proinflammatory lipid implicated in atherosclerosis and inflammatory diseases through its effects on endothelial cells, smooth muscle cells, monocytes, macrophages, and T cells.^45^ In the present study, sphingomyelin and LPC metabolites were inversely associated with the MUFA/SFA ratio and polyunsaturated fatty acid intake, which have been suggested to exert anti-inflammatory and antioxidant effects in the retina.^46^ Ceramide metabolites were also positively associated with fruit juice or sweet consumption, reflecting less healthy dietary patterns. These findings provide potential mechanistic links among diet, lipid metabolism, and AMD pathogenesis. Because these signatures are measured in the circulation, they reflect systemic rather than eye-specific metabolism, and the same signatures have been associated with cardiometabolic outcomes in other populations, including incident type 2 diabetes and cardiovascular disease. Sphingolipid and lysophosphatidylcholine metabolism likewise participate in inflammation, endothelial function, and cellular stress responses across multiple tissues. Whether the associations observed here reflect a shared systemic metabolic milieu that also acts on the retina, or retina-specific effects of these metabolites on photoreceptors and the retinal pigment epithelium, cannot be determined from circulating measurements alone. Studies combining circulating metabolomics with ocular tissue or aqueous and vitreous sampling, and with parallel assessment of extraocular outcomes, would help distinguish these possibilities.

In the MR analyses by incorporating the large-scale AMD genomewide association study (GWAS) summary statistics, genetically instrumented DASH and AHEI-2010 signatures were inversely associated with AMD, whereas the EDIP signature was positively associated. Given the small number of available instruments and their partial overlap with the pleiotropy-prone *FADS1/2* locus, these findings should be interpreted as supportive rather than causal evidence. These findings support the hypothesis that diet-related metabolomic signatures may act as mediators linking diet to AMD development. Interestingly, genetically predicted adherence to AHEI-2010 showed the strongest inverse association with AMD risk, whereas EDIP showed a detrimental association. This discrepancy with our observational findings may reflect limited statistical power in our cross-sectional study or differences between genetically instrumented dietary metabolism and actual dietary intake. Therefore, these results should be interpreted cautiously.

We also explored urinary metabolomic signatures in the subgroup, although significant associations with AMD risk or severity were observed only for PDI, likely due to limited statistical power. Nevertheless, urinary metabolomic signatures may represent a promising noninvasive approach for dietary assessment and warrant further investigation in future studies. Several limitations should be acknowledged. First, the cross-sectional design limits causal inference. Although MR analyses were conducted to explore causality, longitudinal studies are required to confirm these findings. In addition, the MR analyses relied on a small number of genetic instruments (2 to 9 per signature), several of which mapped to the FADS1/2 locus that was itself associated with AMD; horizontal pleiotropy acting through lipid metabolism therefore cannot be excluded. Given the limited instrument count, the pleiotropy-robust estimators (weighted median and mode-based estimation) should be regarded as exploratory, and the IVW estimate was treated as the primary analysis. Second, the study population was predominantly non-Hispanic (91.9%) and, by self-reported race, almost entirely White, with only one Asian and one Black participant, which may limit generalizability to more diverse populations. In addition, participants were recruited from a single tertiary eye center, and controls were drawn from the same clinic population; selection bias and limited generalizability to community-based populations therefore cannot be excluded. In addition, participants with diabetes mellitus were excluded by design. Because diabetes represents the clinical manifestation of sustained hyperinsulinemia, this exclusion is expected to truncate the upper range of insulinemic potential and thereby restrict the variance of the Empirical Dietary Index for Hyperinsulinemia (EDIH) score. Reduced exposure variance would lower statistical power, which may partly explain the null association observed for the EDIH signature, whose association was borderline in the age- and sex-adjusted model. Third, despite adjustment for multiple covariates, residual confounding may remain. Fourth, AMD staging relied on color fundus photography, and fluorescein angiography and OCT angiography were not performed. Exudative disease was therefore identified from intraretinal or subretinal fluid on OCT, which indicates exudation but does not confirm choroidal neovascularization; non-exudative macular neovascularization would not have been detected, and eyes with fluid from other causes could in principle be misclassified. Some misclassification of late AMD is thus possible, although grading was performed independently by two masked graders with adjudication. Relatedly, although OCT images were available, staging relied on color fundus photography alone and OCT was used only to characterize structural features; multimodal imaging can detect lesions, such as subretinal drusenoid deposits and early atrophy that are less apparent on photographs, and may therefore yield somewhat different stage assignments. Finally, metabolomic measurements were obtained at a single time point; repeated measurements would help clarify long-term relationships among diet, metabolomics, and AMD.

## Conclusions

Metabolomic signatures of dietary patterns were associated with AMD risk, disease severity, and OCT-derived retinal structural features, independent of traditional risk factors. These findings highlight the potential of metabolomics to complement conventional dietary assessments and to identify biologically relevant pathways underlying AMD pathogenesis. Future studies are warranted to validate these findings in larger and more diverse populations and to explore their potential clinical applications in AMD prevention and early detection.

## Supplementary Material

Supplement 1

Supplement 2
