# Supplementary material for: Metabolomic Signatures of Healthy Dietary Patterns Associated With Lower Risk of Age-Related Macular Degeneration
Source: Invest Ophthalmol Vis Sci. 2026 Nov 4;67(13):11. doi: 10.1167/iovs.67.13.11 (PMC13743827; doi:10.1167/iovs.67.13.11)
Supplement: Supplementary file 1 [file iovs-67-13-11_s001.docx]

**Supplementary Figure 1. Participant flowcharts**

**Supplementary Figure 2. Distribution of dietary scores and metabolomic signatures.**

**Abbreviations:** AMED, Alternative Mediterranean Diet; AHEI-2010, alternate healthy eating index 2010; DASH, dietary approaches to stop hypertension; EDIH, empirical dietary index for hyperinsulinemia; EDIP, empirical dietary inflammatory pattern; FFQ, food frequency questionnaire; hPDI, healthy plant-based diet index; PDI, plant-based diet index; uPDI, unhealthy plant-based diet index.

**Supplementary Figure 3. Association of plasma metabolomic signatures with severity of age-related macular degeneration in non-Hispanic populations (926 eyes).**

Data is shown as the beta and SE per standard deviation increment in dietary pattern score or metabolomic signature using linear mixed model.

MV: adjusted for age, sex, race, smoking, and BMI.

**Abbreviations:** AMED, Alternative Mediterranean Diet; AHEI-2010, alternate healthy eating index 2010; BMI, body mass index; DASH, dietary approaches to stop hypertension; EDIH, empirical dietary index for hyperinsulinemia; EDIP, empirical dietary inflammatory pattern; hPDI, healthy plant-based diet index; PDI, plant-based diet index; uPDI, unhealthy plant-based diet index.

**Supplementary Figure 4. Stratified analysis of the association of plasma metabolomic signatures with severity of age-related macular degeneration and related structural features.**

**Abbreviations:** AMD, age-related macular degeneration; AMED, Alternative Mediterranean Diet; AHEI-2010, alternate healthy eating index 2010; BMI, body mass index; DASH, dietary approaches to stop hypertension; EDIH, empirical dietary index for hyperinsulinemia; EDIP, empirical dietary inflammatory pattern; EZ, ellipsoid zone; hPDI, healthy plant-based diet index; PDI, plant-based diet index; PED, pigment epithelial detachment; SDD, subretinal drusenoid deposits; uPDI, unhealthy plant-based diet index.

**Supplementary Figure 5. Association of individual metabolites in AMED signature with**

**food components of AMED, AMD severity, and optical coherence tomography features.** Colors denote the association directions (red-positive and blue-inverse); asterisks represent association significance. **P*< 0.05 and **FDR-corrected *P* < 0.05.

**Abbreviations:** AMD, age-related macular degeneration; AMED, Alternative Mediterranean Diet.

**Supplementary Figure 6. Association of individual metabolites in AHEI-2010 signature with food components of AHEI-2010, AMD severity, and optical coherence tomography features.** Colors denote the association directions (red-positive and blue-inverse); asterisks represent association significance. **P*< 0.05 and **FDR-corrected *P* < 0.05.

**Abbreviations:** AHEI-2010, alternate healthy eating index 2010, AMD, age-related macular

Degeneration.

**Supplementary Figure 7. Association of individual metabolites in DASH signature with food components of DASH, AMD severity, and optical coherence tomography features.** Colors denote the association directions (red-positive and blue-inverse); asterisks represent association significance. **P*< 0.05 and **FDR-corrected *P* < 0.05.

**Abbreviations:** AMD, age-related macular degeneration; DASH, dietary approaches to stop hypertension.

**Supplementary Figure 8. Association of individual metabolites in PDI signature with food components of PDI, AMD severity, and optical coherence tomography features.** Colors denote the association directions (red-positive and blue-inverse); asterisks represent association significance. **P*< 0.05 and **FDR-corrected *P* < 0.05.

**Abbreviations:** AMD, age-related macular degeneration; PDI, plant-based diet index.

**Supplementary Figure 9. Association of individual metabolites in hPDI signature with food components of hPDI, AMD severity, and optical coherence tomography features.** Colors denote the association directions (red-positive and blue-inverse); asterisks represent association significance. **P*< 0.05 and **FDR-corrected *P* < 0.05.

**Abbreviations:** AMD, age-related macular degeneration; hPDI, healthy plant-based diet index.

**Supplementary Figure 10. Association of individual metabolites in uPDI signature with food components of uPDI, AMD severity, and optical coherence tomography features.** Colors denote the association directions (red-positive and blue-inverse); asterisks represent association significance. **P*< 0.05 and **FDR-corrected *P* < 0.05.

**Abbreviations:** AMD, age-related macular degeneration; uPDI, unhealthy plant-based diet index.

**Supplementary Figure 11. Association of individual metabolites in EDIP signature with food components of EDIP, AMD severity, and optical coherence tomography features.** Colors denote the association directions (red-positive and blue-inverse); asterisks represent association significance. **P*< 0.05 and **FDR-corrected *P* < 0.05.

**Abbreviations:** AMD, age-related macular degeneration; EDIP, empirical dietary inflammatory pattern.

**Supplementary Figure 12. Association of individual metabolites in EDIH signature with food components of EDIH, AMD severity, and optical coherence tomography features.** Colors denote the association directions (red-positive and blue-inverse); asterisks represent association significance. **P*< 0.05 and **FDR-corrected *P* < 0.05.

**Abbreviations:** AMD, age-related macular degeneration; EDIH, empirical dietary index for hyperinsulinemia.

**Supplementary Figure 13. Genetically inferred metabolomic signatures and risk of** age-related macular degeneration**.**

**Supplementary Figure 14. Genetically inferred metabolomic signatures and risk of age-related macular degeneration.**

**Abbreviations:** AMED, Alternative Mediterranean Diet; AHEI-2010, alternate healthy eating index 2010; DASH, dietary approaches to stop hypertension; EDIH, empirical dietary index for hyperinsulinemia; EDIP, empirical dietary inflammatory pattern; hPDI, healthy plant-based diet index; PDI, plant-based diet index; uPDI, unhealthy plant-based diet index.

**Supplementary Table 1. Number of available metabolites composing of the metabolomic signature in the study population**

| **Trait** | **No. of metabolites (Raw model)** | **No. of**  **metabolites (Available in plasma)** | **No. of**  **metabolites (Available in urine)** |
| --- | --- | --- | --- |
| AMED | 48 | 38 | 33 |
| AHEI-2010 | 61 | 54 | 41 |
| DASH | 55 | 45 | 36 |
| PDI | 36 | 26 | 26 |
| hPDI | 39 | 30 | 26 |
| uPDI | 43 | 35 | 31 |
| EDIP | 66 | 54 | 47 |
| EDIH | 37 | 33 | 23 |

**Abbreviations:** AMED, Alternative Mediterranean Diet; AHEI-2010, alternate healthy eating index 2010; DASH, dietary approaches to stop hypertension; EDIH, empirical dietary index for hyperinsulinemia; EDIP, empirical dietary inflammatory pattern; hPDI, healthy plant-based diet index; PDI, plant-based diet index; uPDI, unhealthy plant-based diet index

**Supplemental Table 2. Baseline characteristics of the subpopulation with both plasma and urine metabolomic signatures (400 eyes of 200 participants)**

| **Characteristic** | **Normal**  **(96 eyes)** | **Early AMD**  **(63 eyes)** | **Intermediate AMD**  **(134 eyes)** | **Advanced AMD**  **(67 eyes)** | ***P*_overall_** |
| --- | --- | --- | --- | --- | --- |
| Age, years | 68.6 (7.95) | 70.3 (8.69) | 72.0 (7.43) | 76.0 (7.78) | 0.001 |
| Male, *n* (%) | 23 (44.2%) | 7 (24.1%) | 30 (40.5%) | 8 (33.3%) | 0.302 |
| **Race/Ethnicity, *n* (%)** |  |  |  |  | 0.267 |
| Non-Hispanic | 51 (98.1%) | 25 (86.2%) | 71 (95.9%) | 23 (95.8%) |  |
| Hispanic | 1 (1.92%) | 2 (6.90%) | 2 (2.70%) | 1 (4.17%) |  |
| Others | 0 (0.00%) | 2 (6.90%) | 1 (1.35%) | 0 (0.00%) |  |
| **Smoking status, *n* (%)** |  |  |  |  | 0.377 |
| Never | 27 (51.9%) | 17 (58.6%) | 31 (41.9%) | 8 (33.3%) |  |
| Current smoker | 2 (3.85%) | 0 (0.00%) | 3 (4.05%) | 0 (0.00%) |  |
| Previous smoker | 23 (44.2%) | 12 (41.4%) | 40 (54.1%) | 16 (66.7%) |  |
| BMI, kg/m^2^ | 27.4 (5.62) | 26.8 (4.86) | 26.8 (4.38) | 27.8 (4.79) | 0.800 |
| **Dietary pattern score** |  |  |  |  |  |
| AMED | 4.54 (4.28, 4.88) | 4.5 (4.25, 4.67) | 4.64 (4.35, 4.92) | 4.56 (4.24, 4.86) | 0.328 |
| AHEI-2010 | 68.54 (65.5, 72.03) | 70.02 (65.22, 71.91) | 69.18 (66.96, 71.92) | 68.68 (66.15, 71.04) | 0.792 |
| DASH | 24.2 (23.62, 25.48) | 24.02 (23.15, 25) | 24.23 (23.31, 25.2) | 24.1 (23.08, 24.98) | 0.623 |
| PDI | 53.33 (52.75, 54.14) | 53.33 (52.52, 53.84) | 53.2 (52.93, 53.91) | 53.11 (51.86, 53.73) | 0.178 |
| hPDI | 54.39 (53.22, 55.45) | 54.43 (52.94, 55.01) | 54.58 (53.4, 55.83) | 54.89 (53.39, 55.49) | 0.228 |
| uPDI | 53.98 (52.67, 55.84) | 53.03 (52.25, 55.62) | 53.63 (51.8, 55.14) | 53.42 (51.83, 55.9) | 0.525 |
| EDIP | -0.13 (-0.23, -0.03) | -0.09 (-0.2, -0.01) | -0.14 (-0.24, 0) | -0.15 (-0.19, -0.07) | 0.830 |
| EDIH | 0.38 (0.34, 0.42) | 0.40 (0.36, 0.43) | 0.38 (0.35, 0.43) | 0.39 (0.35, 0.42) | 0.476 |

Data is presented as mean (SD) or median (IQR) for continuous variables and *n* (percentage) for categorical variables.

**Abbreviations:** AMD, age-related macular degeneration; AMED, Alternative Mediterranean Diet; AHEI-2010, alternate healthy eating index 2010; BMI, body mass index; DASH, dietary approaches to stop hypertension; EDIH, empirical dietary index for hyperinsulinemia; EDIP, empirical dietary inflammatory pattern; hPDI, healthy plant-based diet index; IQR, interquartile range; PDI, plant-based diet index; SD, standard deviation; uPDI, unhealthy plant-based diet index.

**Supplemental Table 3. Association of urinary metabolomic signatures with risk of age-related macular degeneration^1^**

|  | **Model1^2^** | | **Model2^3^** | |
| --- | --- | --- | --- | --- |
|  | **OR (95% CI)** | ***P*** | **OR (95% CI)** | ***P*** |
| **Dietary pattern score** | |  |  |  |
| AMED | 0.95 (0.40,2.25) | 0.910 | 0.96 (0.86,1.07) | 0.462 |
| AHEI-2010 | 0.88 (0.37,2.11) | 0.775 | 0.92 (0.83,1.03) | 0.135 |
| DASH | 0.95 (0.39,2.33) | 0.911 | 0.99 (0.89,1.09) | 0.807 |
| PDI | 0.93 (0.37,2.30) | 0.868 | 0.97 (0.88,1.08) | 0.611 |
| hPDI | 0.85 (0.35,2.08) | 0.725 | 0.94 (0.85,1.05) | 0.282 |
| uPDI | 1.05 (0.44,2.51) | 0.913 | 0.97 (0.87,1.07) | 0.528 |
| EDIP | 0.97 (0.41,2.32) | 0.948 | 1.00 (0.90,1.10) | 0.971 |
| EDIH | 1.03 (0.43,2.46) | 0.944 | 1.00 (0.91,1.11) | 0.924 |
| **Metabolomic signature** | | |  |  |
| AMED | 0.96 (0.24,3.90) | 0.956 | 1.00 (0.85,1.17) | 0.959 |
| AHEI-2010 | 1.00 (0.23,4.35) | 1.00 | 1.02 (0.86,1.20) | 0.817 |
| DASH | 0.79 (0.19,3.32) | 0.753 | 0.94 (0.80,1.10) | 0.437 |
| PDI | 0.67 (0.17,2.63) | 0.561 | 0.85 (0.73,1.00) | **0.048** |
| hPDI | 1.07 (0.29,3.93) | 0.924 | 1.07 (0.92,1.26) | 0.388 |
| uPDI | 0.95 (0.24,3.81) | 0.943 | 0.94 (0.80,1.11) | 0.477 |
| EDIP | 1.25 (0.33,4.70) | 0.744 | 1.00 (0.85,1.18) | 0.977 |
| EDIH | 1.25 (0.35,4.41) | 0.728 | 1.04 (0.89,1.22) | 0.592 |

^1^Odds ratio (OR) and 95% confidence interval (CI) of AMD severity per standard deviation increment in dietary pattern score or metabolomic signature using linear mixed model.

^2^Model 1: adjusted for age, sex.

^3^Model 2: further adjusted for race, smoking, and BMI.

**Abbreviations:** AMED, Alternative Mediterranean Diet; BMI, body mass index; AHEI-2010, alternate healthy eating index 2010; DASH, dietary approaches to stop hypertension; EDIH, empirical dietary index for hyperinsulinemia; EDIP, empirical dietary inflammatory pattern; hPDI, healthy plant-based diet index; PDI, plant-based diet index; uPDI, unhealthy plant-based diet index.

**Supplemental Table 4. Stratified analysis of the association between plasma metabolomic signatures and severity of** **age-related macular degeneration**

| **Metabolomic signature** | **Group** | **N** | **Est** | **SE** | ***P*** | ***P*_interaction_** |
| --- | --- | --- | --- | --- | --- | --- |
| **Age** | | | | | | |
| **AMED** | <65 y | 182 | -0.144 | 0.159 | 0.364 | 0.660 |
|  | ≥65 y | 826 | -0.134 | 0.059 | **0.022** |  |
| **AHEI-2010** | <65 y | 182 | 0.091 | 0.161 | 0.571 | 0.286 |
|  | ≥65 y | 826 | -0.106 | 0.059 | 0.0695 |  |
| **DASH** | <65 y | 182 | 0.030 | 0.164 | 0.855 | 0.045 |
|  | ≥65 y | 826 | -0.207 | 0.058 | **<0.0001** |  |
| **PDI** | <65 y | 182 | -0.022 | 0.140 | 0.875 | 0.154 |
|  | ≥65 y | 826 | -0.195 | 0.060 | **0.001** |  |
| **hPDI** | <65 y | 182 | -0.009 | 0.153 | 0.952 | 0.175 |
|  | ≥65 y | 826 | -0.186 | 0.058 | **0.001** |  |
| **uPDI** | <65 y | 182 | 0.164 | 0.140 | 0.241 | 0.821 |
|  | ≥65 y | 826 | 0.119 | 0.056 | **0.034** |  |
| **EDIP** | <65 y | 182 | -0.148 | 0.128 | 0.250 | 0.105 |
|  | ≥65 y | 826 | -0.033 | 0.057 | 0.567 |  |
| **EDIH** | <65 y | 182 | -0.095 | 0.147 | 0.518 | 0.240 |
|  | >=65 y | 826 | 0.089 | 0.057 | 0.120 |  |
| **Sex** | | | | | | |
| **AMED** | Male | 370 | -0.177 | 0.103 | 0.086 | 0.596 |
|  | Female | 638 | -0.116 | 0.063 | 0.063 |  |
| **AHEI-2010** | Male | 370 | -0.136 | 0.100 | 0.172 | 0.101 |
|  | Female | 638 | -0.065 | 0.066 | 0.326 |  |
| **DASH** | Male | 370 | -0.196 | 0.100 | **0.050** | 0.212 |
|  | Female | 638 | -0.172 | 0.065 | **0.008** |  |
| **PDI** | Male | 370 | -0.171 | 0.090 | 0.059 | 0.447 |
|  | Female | 638 | -0.167 | 0.069 | **0.015** |  |
| **hPDI** | Male | 370 | -0.127 | 0.103 | 0.219 | 0.511 |
|  | Female | 638 | -0.163 | 0.063 | **0.010** |  |
| **uPDI** | Male | 370 | 0.120 | 0.096 | 0.211 | 0.666 |
|  | Female | 638 | 0.131 | 0.061 | **0.032** |  |
| **EDIP** | Male | 370 | -0.051 | 0.086 | 0.556 | 0.0654 |
|  | Female | 638 | -0.074 | 0.064 | 0.248 |  |
| **EDIH** | Male | 370 | 0.052 | 0.093 | 0.577 | 0.0567 |
|  | Female | 638 | 0.043 | 0.064 | 0.499 |  |
| **Smoking status** | | | | | | |
| **AMED** | Never | 480 | -0.131 | 0.076 | 0.083 | 0.497 |
|  | Former | 524 | -0.149 | 0.075 | **0.048** |  |
| **AHEI-2010** | Never | 480 | -0.089 | 0.079 | 0.258 | 0.0884 |
|  | Former | 524 | -0.066 | 0.076 | 0.387 |  |
| **DASH** | Never | 480 | -0.196 | 0.079 | **0.013** | 0.232 |
|  | Former | 524 | -0.171 | 0.074 | **0.020** |  |
| **PDI** | Never | 480 | -0.152 | 0.079 | 0.053 | 0.409 |
|  | Former | 524 | -0.172 | 0.075 | **0.022** |  |
| **hPDI** | Never | 480 | -0.145 | 0.083 | 0.082 | 0.563 |
|  | Former | 524 | -0.169 | 0.069 | **0.015** |  |
| **uPDI** | Never | 480 | 0.175 | 0.075 | **0.020** | 0.823 |
|  | Former | 524 | 0.064 | 0.070 | 0.359 |  |
| **EDIP** | Never | 480 | 0.017 | 0.075 | 0.816 | 0.114 |
|  | Former | 524 | -0.121 | 0.070 | 0.083 |  |
| **EDIH** | Never | 480 | 0.114 | 0.079 | 0.149 | 0.0666 |
|  | Former | 524 | 0.012 | 0.071 | 0.864 |  |
| **BMI** | | | | | | |
| **AMED** | <25 kg/m^2^ | 296 | -0.173 | 0.084 | **0.040** | 0.448 |
|  | ≥25 kg/m^2^ | 476 | -0.082 | 0.071 | 0.248 |  |
| **AHEI-2010** | <25 kg/m^2^ | 296 | -0.114 | 0.085 | 0.180 | 0.0974 |
|  | ≥25 kg/m^2^ | 476 | -0.041 | 0.072 | 0.575 |  |
| **DASH** | <25 kg/m^2^ | 296 | -0.187 | 0.082 | **0.022** | 0.290 |
|  | ≥25 kg/m^2^ | 476 | -0.157 | 0.074 | **0.034** |  |
| **PDI** | <25 kg/m^2^ | 296 | -0.140 | 0.091 | 0.125 | 0.503 |
|  | ≥25 kg/m^2^ | 476 | -0.170 | 0.071 | **0.017** |  |
| **hPDI** | <25 kg/m^2^ | 296 | -0.128 | 0.080 | 0.110 | 0.554 |
|  | ≥25 kg/m^2^ | 476 | -0.154 | 0.074 | **0.037** |  |
| **uPDI** | <25 kg/m^2^ | 296 | 0.103 | 0.081 | 0.201 | 0.518 |
|  | ≥25 kg/m^2^ | 476 | 0.123 | 0.068 | 0.072 |  |
| **EDIP** | <25 kg/m^2^ | 296 | -0.052 | 0.084 | 0.535 | 0.0686 |
|  | ≥25 kg/m^2^ | 476 | -0.055 | 0.065 | 0.400 |  |
| **EDIH** | <25 kg/m^2^ | 296 | 0.008 | 0.085 | 0.926 | 0.0743 |
|  | ≥25 kg/m^2^ | 476 | 0.078 | 0.068 | 0.253 |  |

**Abbreviations:** AMED, Alternative Mediterranean Diet; AHEI-2010, alternate healthy eating index 2010; BMI, body mass index; DASH, dietary approaches to stop hypertension; EDIH, empirical dietary index for hyperinsulinemia; EDIP, empirical dietary inflammatory pattern; hPDI, healthy plant-based diet index; PDI, plant-based diet index; uPDI, unhealthy plant-based diet index.

**Supplemental Table 5. Association between urinary metabolomic signatures and severity of age-related macular degeneration ^1^**

|  | **Model1^2^** | | | **Model2^3^** | | | **Model3^4^** | | |
| --- | --- | --- | --- | --- | --- | --- | --- | --- | --- |
|  | **Beta** | **SE** | ***P*** | **Beta** | **SE** | ***P*** | **Beta** | **SE** | ***P*** |
| **Dietary pattern score** | |  |  |  |  |  |  |  |  |
| AMED | -0.058 | 0.046 | 0.210 | -0.041 | 0.056 | 0.461 | -0.007 | 0.058 | 0.897 |
| AHEI-2010 | -0.094 | 0.045 | **0.037** | -0.080 | 0.053 | 0.136 | -0.069 | 0.054 | 0.206 |
| DASH | -0.039 | 0.045 | 0.384 | -0.013 | 0.052 | 0.806 | 0.024 | 0.053 | 0.653 |
| PDI | -0.035 | 0.045 | 0.431 | -0.027 | 0.052 | 0.609 | -0.002 | 0.053 | 0.974 |
| hPDI | -0.067 | 0.045 | 0.137 | -0.058 | 0.055 | 0.284 | -0.034 | 0.055 | 0.539 |
| uPDI | -0.020 | 0.045 | 0.654 | -0.033 | 0.053 | 0.530 | -0.056 | 0.054 | 0.298 |
| EDIP | 0.031 | 0.045 | 0.489 | -0.002 | 0.052 | 0.971 | 0.002 | 0.176 | 0.990 |
| EDIH | 0.048 | 0.045 | 0.286 | 0.005 | 0.051 | 0.924 | -0.057 | 0.185 | 0.757 |
| **Metabolomic signature** | | |  |  |  |  |  |  |  |
| AMED | -0.041 | 0.077 | 0.598 | -0.004 | 0.082 | 0.959 | 0.036 | 0.085 | 0.669 |
| AHEI-2010 | -0.026 | 0.076 | 0.729 | 0.019 | 0.084 | 0.817 | -0.039 | 0.086 | 0.652 |
| DASH | -0.097 | 0.076 | 0.199 | -0.063 | 0.081 | 0.437 | -0.039 | 0.086 | 0.652 |
| PDI | -0.195 | 0.075 | **0.009*** | -0.157 | 0.079 | **0.048** | -0.139 | 0.080 | 0.083 |
| hPDI | 0.043 | 0.076 | 0.577 | 0.070 | 0.081 | 0.388 | 0.111 | 0.082 | 0.178 |
| uPDI | -0.026 | 0.078 | 0.740 | -0.060 | 0.084 | 0.477 | -0.089 | 0.089 | 0.314 |
| EDIP | 0.009 | 0.077 | 0.912 | 0.002 | 0.082 | 0.977 | -0.048 | 0.085 | 0.575 |
| EDIH | 0.053 | 0.076 | 0.483 | 0.043 | 0.080 | 0.592 | -0.007 | 0.085 | 0.935 |

^1^Data is presented as beta and SE per standard deviation increment in dietary pattern score or metabolomic signature using linear mixed model.

^2^Model 1: adjusted for age, sex.

^3^Model 2: further adjusted for race, smoking, and BMI.

^4^Model 3: both dietary index and corresponding metabolomic signature were simultaneously included to examine association independence.

**Abbreviations:** AMED, Alternative Mediterranean Diet; BMI, body mass index; AHEI-2010, alternate healthy eating index 2010; DASH, dietary approaches to stop hypertension; EDIH, empirical dietary index for hyperinsulinemia; EDIP, empirical dietary inflammatory pattern; hPDI, healthy plant-based diet index; PDI, plant-based diet index; uPDI, unhealthy plant-based diet index.

**Supplemental Table 6. Characteristics of genetic instruments for dietary metabolomic signatures**

| **Trait** | **SNP** | **Nearest gene** | **CHR** | **Position** | **A1** | **A2** | **Exposure^1^** | | | **AMD^2^** | | |
| --- | --- | --- | --- | --- | --- | --- | --- | --- | --- | --- | --- | --- |
|  |  |  |  |  |  |  | **Beta** | **SE** | ***P*** | **Beta** | **SE** | ***P*** |
| AMED | rs12785109 | *RP11-56M3.1* | 10 | 92931758 | T | C | 0.006 | 0.014 | 2.09E-08 | 0.001 | 0.017 | 9.76E-01 |
|  | rs174553 | *FADS1/2* | 11 | 61575158 | G | A | -0.212 | 0.012 | 3.41E-72 | 0.054 | 0.018 | 1.94E-03 |
|  | rs174455 | *FADS3* | 11 | 61656117 | A | G | -0.114 | 0.014 | 4.74E-16 | -0.013 | 0.018 | 4.48E-01 |
|  | rs61940387 | *RP11-955H22.2* | 12 | 128067147 | G | A | -0.024 | 0.020 | 4.49E-08 | 0.012 | 0.027 | 6.62E-01 |
|  | rs12318075 | *SLCO1B1* | 12 | 21361587 | G | T | 0.101 | 0.018 | 5.24E-11 | 0.003 | 0.022 | 8.88E-01 |
|  | rs202932 | *RP11-384J4.2* | 14 | 27684014 | G | C | -0.033 | 0.014 | 1.24E-08 | -0.027 | 0.018 | 1.27E-01 |
|  | rs2036815 | *MIR4510* | 15 | 36230632 | T | C | 0.005 | 0.014 | 3.18E-08 | 0.003 | 0.022 | 8.89E-01 |
|  | rs159311 | *CCL1* | 17 | 32712649 | T | C | 0.010 | 0.019 | 5.64E-09 | 0.016 | 0.023 | 4.85E-01 |
|  | rs148858632 | *RP11-879F14.1* | 18 | 59414086 | C | T | -0.083 | 0.051 | 3.13E-08 | 0.008 | 0.079 | 9.14E-01 |
|  | rs35779543 | *AC016751.3* | 2 | 176765645 | C | T | 0.036 | 0.022 | 9.87E-09 | 0.005 | 0.035 | 8.83E-01 |
|  | rs1047891 | *CPS1* | 2 | 211540507 | A | C | -0.077 | 0.015 | 1.31E-08 | 0.033 | 0.019 | 8.73E-02 |
|  | rs11903916 | *ALMS1* | 2 | 73815328 | G | A | -0.106 | 0.012 | 2.14E-20 | 0.005 | 0.019 | 7.81E-01 |
|  | rs11689234 | *AC092653.5* | 2 | 73931716 | T | C | -0.102 | 0.016 | 2.49E-10 | 0.016 | 0.020 | 4.12E-01 |
|  | rs17138596 | *CAV2* | 7 | 115955097 | C | T | 0.111 | 0.021 | 4.46E-08 | -0.017 | 0.025 | 5.11E-01 |
| AHEI-2010 | rs12360725 | *OR5AM1P* | 11 | 56395892 | T | G | 0.060 | 0.012 | 4.26E-08 | 0.037 | 0.020 | 6.13E-02 |
|  | rs174553 | *FADS1/2* | 11 | 61575158 | G | A | -0.229 | 0.011 | 2.91E-95 | -0.054 | 0.018 | 1.94E-03 |
|  | rs174455 | *FADS3* | 11 | 61656117 | A | G | -0.128 | 0.014 | 2.65E-20 | 0.013 | 0.018 | 4.48E-01 |
|  | rs659122 | *C11orf85* | 11 | 64738420 | A | G | -0.034 | 0.011 | 1.62E-08 | -0.013 | 0.017 | 4.55E-01 |
|  | rs2512468 | *CTD-2555I5.1* | 11 | 80171021 | A | G | 0.027 | 0.018 | 1.72E-08 | -0.039 | 0.024 | 1.02E-01 |
|  | rs11612057 | *H2AFJ* | 12 | 14929642 | T | A | -0.074 | 0.017 | 8.26E-09 | -0.018 | 0.020 | 3.71E-01 |
|  | rs57743625 | *SLCO1B1* | 12 | 21367633 | A | G | -0.080 | 0.018 | 7.85E-09 | -0.002 | 0.022 | 9.15E-01 |
|  | rs6571997 | *RP11-111A21.1* | 14 | 40783583 | C | G | -0.021 | 0.015 | 9.01E-10 | -0.011 | 0.018 | 5.46E-01 |
|  | rs2678790 | *RP11-784B15.1* | 18 | 28180154 | T | C | 0.007 | 0.025 | 1.42E-08 | 0.001 | 0.030 | 9.65E-01 |
|  | rs60541386 | *SNAI1* | 20 | 48602508 | C | T | -0.027 | 0.020 | 7.38E-11 | -0.008 | 0.025 | 7.40E-01 |
|  | rs11921336 | *TPRG1* | 3 | 188883104 | G | A | -0.017 | 0.027 | 4.98E-08 | 0.012 | 0.036 | 7.52E-01 |
|  | rs3095870 | *NKX2-5* | 5 | 172663454 | T | C | -0.040 | 0.014 | 3.83E-08 | -0.027 | 0.018 | 1.32E-01 |
| DASH | rs174553 | *FADS1/2* | 11 | 61575158 | G | A | -0.210 | 0.012 | 4.55E-68 | 0.054 | 0.018 | 1.94E-03 |
|  | rs174455 | *FADS3* | 11 | 61656117 | A | G | -0.111 | 0.014 | 9.13E-16 | -0.013 | 0.018 | 4.48E-01 |
|  | rs11045892 | *SLCO1B1* | 12 | 21392794 | G | A | -0.069 | 0.017 | 2.35E-08 | -0.001 | 0.021 | 9.66E-01 |
|  | rs11045909 | *SLCO1B1* | 12 | 21411206 | G | C | 0.096 | 0.018 | 1.19E-11 | -0.001 | 0.022 | 9.77E-01 |
|  | rs2617921 | *RP11-784B15.1* | 18 | 28178010 | T | C | -0.009 | 0.024 | 1.52E-08 | -0.001 | 0.030 | 9.62E-01 |
|  | rs11899902 | *ALMS1* | 2 | 73631564 | A | G | -0.095 | 0.013 | 5.04E-14 | -0.008 | 0.020 | 7.00E-01 |
|  | rs11689234 | *AC092653.5* | 2 | 73931716 | T | C | -0.090 | 0.016 | 1.65E-08 | 0.016 | 0.020 | 4.12E-01 |
|  | rs55649827 | *RP5-1022P6.4* | 20 | 5508885 | A | T | -0.016 | 0.018 | 4.40E-08 | 0.014 | 0.023 | 5.34E-01 |
|  | rs57260240 | *PPARGC1A* | 4 | 23870248 | G | C | -0.042 | 0.046 | 3.54E-09 | -0.020 | 0.058 | 7.34E-01 |
|  | rs75997818 | *FYN* | 6 | 112013310 | G | C | -0.039 | 0.034 | 3.25E-08 | -0.052 | 0.042 | 2.19E-01 |
| PDI | rs188413635 | *ST7L* | 1 | 113121421 | A | C | -0.053 | 0.053 | 3.10E-08 | 0.045 | 0.077 | 5.57E-01 |
|  | rs1922392 | *NAV2* | 11 | 19928311 | A | G | 0.023 | 0.021 | 1.30E-08 | 0.033 | 0.038 | 3.86E-01 |
|  | rs174553 | *FADS1/2* | 11 | 61575158 | G | A | -0.140 | 0.013 | 1.17E-28 | -0.054 | 0.018 | 1.94E-03 |
|  | rs174476 | *RAB3IL1* | 11 | 61674118 | T | C | -0.080 | 0.014 | 7.05E-09 | 0.016 | 0.017 | 3.58E-01 |
|  | rs11607200 | *GRM5* | 11 | 88257930 | C | T | -0.009 | 0.020 | 8.23E-09 | -0.054 | 0.026 | 3.57E-02 |
|  | rs11042497 | *SBF2-AS1* | 11 | 9815208 | C | G | -0.005 | 0.014 | 2.45E-08 | 0.019 | 0.017 | 2.74E-01 |
|  | rs112553770 | *RP11-184D12.1* | 15 | 36546088 | C | T | -0.016 | 0.028 | 1.28E-08 | -0.038 | 0.043 | 3.75E-01 |
|  | rs2650424 | *GRIN2A* | 16 | 10091753 | A | T | 0.043 | 0.017 | 3.16E-08 | 0.069 | 0.020 | 5.75E-04 |
|  | rs35451178 | *PIAS2* | 18 | 44407993 | A | G | 0.097 | 0.044 | 2.34E-08 | -0.011 | 0.059 | 8.58E-01 |
|  | rs7253890 | *CERS4* | 19 | 8272775 | T | A | -0.117 | 0.015 | 1.14E-15 | -0.011 | 0.018 | 5.50E-01 |
|  | rs12485974 | *FAM86JP* | 3 | 125644009 | A | G | 0.082 | 0.017 | 1.34E-08 | 0.024 | 0.026 | 3.51E-01 |
|  | rs17037105 | *DKK2* | 4 | 107846116 | C | T | 0.035 | 0.027 | 2.43E-08 | -0.039 | 0.033 | 2.32E-01 |
| hPDI | rs174553 | *FADS1* | 11 | 61575158 | G | A | -0.092 | 0.012 | 1.12E-14 | 0.054 | 0.018 | 1.94E-03 |
|  | rs1470053 | *BCL2L11* | 2 | 111915946 | T | G | -0.052 | 0.017 | 2.83E-08 | -0.029 | 0.023 | 1.98E-01 |
|  | rs71443098 | *WDR43* | 2 | 29129712 | T | G | -0.023 | 0.016 | 2.41E-08 | 0.036 | 0.020 | 6.91E-02 |
|  | rs62149622 | *None* | 2 | 73535292 | C | A | 0.157 | 0.017 | 1.27E-21 | 0.006 | 0.025 | 8.00E-01 |
|  | rs4547554 | *ALMS1* | 2 | 73867862 | C | G | -0.196 | 0.013 | 9.69E-53 | -0.010 | 0.020 | 6.01E-01 |
|  | rs56055081 | *STAMBP* | 2 | 74086025 | A | C | -0.150 | 0.030 | 4.74E-09 | -0.036 | 0.041 | 3.85E-01 |
|  | rs6118809 | *PAK7* | 20 | 9901312 | T | C | 0.072 | 0.015 | 3.05E-08 | -0.007 | 0.018 | 6.86E-01 |
|  | rs74280921 | *PROK2* | 3 | 71873138 | T | C | -0.049 | 0.033 | 3.43E-09 | 0.028 | 0.053 | 5.92E-01 |
| uPDI | rs3887698 | *TACSTD2* | 1 | 59056733 | A | T | -0.001 | 0.015 | 4.32E-08 | 0.022 | 0.016 | 1.74E-01 |
|  | rs174561 | *FADS1/2* | 11 | 61582708 | C | T | 0.108 | 0.012 | 2.37E-21 | -0.051 | 0.018 | 5.04E-03 |
|  | rs7969341 | *SLCO1B1* | 12 | 21385604 | G | A | -0.060 | 0.014 | 1.83E-08 | -0.002 | 0.022 | 9.37E-01 |
|  | rs62039719 | *UNGP1* | 16 | 51328159 | C | T | -0.195 | 0.036 | 2.18E-09 | -0.055 | 0.053 | 2.94E-01 |
|  | rs17259154 | *TMEM241* | 18 | 20889611 | C | T | -0.079 | 0.020 | 4.91E-08 | 0.041 | 0.050 | 4.10E-01 |
|  | rs6507582 | *SETBP1* | 18 | 42365242 | A | T | 0.021 | 0.015 | 4.95E-08 | -0.006 | 0.019 | 7.60E-01 |
|  | rs35293936 | *PTH2R* | 2 | 209227348 | T | C | -0.010 | 0.017 | 2.23E-08 | -0.016 | 0.026 | 5.30E-01 |
|  | rs17349049 | *None* | 2 | 73529077 | A | G | 0.097 | 0.017 | 1.07E-08 | -0.008 | 0.026 | 7.71E-01 |
|  | rs10198549 | *ALMS1* | 2 | 73794404 | G | A | -0.112 | 0.012 | 2.66E-21 | 0.004 | 0.020 | 8.29E-01 |
|  | rs113566031 | *STAMBP* | 2 | 74089028 | G | A | -0.081 | 0.024 | 2.93E-08 | 0.024 | 0.032 | 4.45E-01 |
|  | rs2540656 | *DGCR5* | 22 | 18974973 | T | C | 0.096 | 0.023 | 3.58E-09 | -0.006 | 0.029 | 8.36E-01 |
|  | rs1553455 | *ABI3BP* | 3 | 100480196 | A | C | 0.021 | 0.016 | 1.93E-08 | 0.034 | 0.019 | 7.93E-02 |
|  | rs62277509 | *AC092965.1* | 3 | 166769640 | T | G | -0.001 | 0.015 | 4.19E-09 | 0.031 | 0.018 | 8.91E-02 |
|  | rs114694736 | *CCSER1* | 4 | 92113737 | C | T | 0.056 | 0.051 | 2.60E-08 | -0.083 | 0.068 | 2.22E-01 |
|  | rs6875488 | *CDH12* | 5 | 22585662 | C | A | -0.042 | 0.015 | 3.98E-08 | -0.005 | 0.019 | 7.72E-01 |
|  | rs12654485 | *PDE4D* | 5 | 58407448 | G | A | -0.017 | 0.030 | 4.24E-08 | 0.030 | 0.040 | 4.55E-01 |
|  | rs74510798 | *RP11-307L14.2* | 5 | 64373164 | A | G | 0.081 | 0.040 | 9.32E-09 | -0.017 | 0.058 | 7.66E-01 |
|  | rs17138596 | *AC073130.3* | 7 | 115955097 | C | T | -0.094 | 0.021 | 1.36E-08 | 0.017 | 0.025 | 5.11E-01 |
| EDIP | rs7094248 | *CHAT* | 10 | 50855368 | G | C | -0.014 | 0.014 | 4.71E-09 | -0.003 | 0.017 | 8.50E-01 |
|  | rs111966496 | *PRR5L* | 11 | 36461930 | T | C | -0.120 | 0.053 | 2.75E-08 | 0.073 | 0.076 | 3.40E-01 |
|  | rs174561 | *FADS1/2* | 11 | 61582708 | C | T | 0.063 | 0.012 | 4.23E-08 | 0.051 | 0.018 | 5.04E-03 |
|  | rs59794053 | *MMP14* | 14 | 23321112 | A | G | 0.010 | 0.019 | 2.11E-08 | 0.036 | 0.025 | 1.59E-01 |
|  | rs1367344 | *RN7SL401P* | 18 | 71479072 | C | T | -0.015 | 0.017 | 4.80E-08 | -0.049 | 0.026 | 5.39E-02 |
|  | rs6045630 | *C20orf78* | 20 | 18795890 | A | T | -0.021 | 0.029 | 4.56E-08 | -0.026 | 0.036 | 4.58E-01 |
|  | rs59245017 | *RP5-1043L13.1* | 20 | 58982212 | A | G | -0.029 | 0.014 | 2.60E-08 | -0.019 | 0.017 | 2.80E-01 |
|  | rs7695576 | *AC092846.2* | 4 | 24436960 | A | G | -0.051 | 0.015 | 3.29E-08 | -0.014 | 0.019 | 4.47E-01 |
| EDIH | rs174538 | *TMEM258* | 11 | 61560081 | A | G | -0.068 | 0.012 | 2.51E-10 | -0.051 | 0.018 | 4.38E-03 |
|  | rs2147006 | *RP11-29G8.3* | 13 | 76244056 | G | A | 0.032 | 0.018 | 4.31E-08 | -0.012 | 0.023 | 6.05E-01 |
|  | rs9708471 | *CDH13* | 16 | 83394808 | G | A | -0.059 | 0.015 | 3.61E-08 | 0.002 | 0.019 | 8.96E-01 |
|  | rs2357157 | *YWHAQ* | 2 | 9758217 | A | G | -0.082 | 0.015 | 9.84E-09 | 0.012 | 0.018 | 5.12E-01 |
|  | rs1683776 | *ACAD9* | 3 | 128622760 | T | G | 0.025 | 0.016 | 4.14E-08 | -0.020 | 0.020 | 3.16E-01 |
|  | rs35380575 | *snoZ40* | 3 | 86277963 | G | C | -0.041 | 0.022 | 4.10E-08 | 0.012 | 0.027 | 6.47E-01 |

^1^Summary data for dietary metabolomic signatures was from our recent publication (n=13,700).

^2^Summary data for AMD was from International AMD Genomics Consortium (16,144 late-stage AMD cases and 17,832 AMD-free controls).

**Abbreviations:** AMD, age-related macular degeneration; AMED, Alternative Mediterranean Diet; AHEI-2010, alternate healthy eating index 2010; Chr, chromosome; DASH, dietary approaches to stop hypertension; EDIH, empirical dietary index for hyperinsulinemia; EDIP, empirical dietary inflammatory pattern; hPDI, healthy plant-based diet index; PDI, plant-based diet index; SNP, single nucleotide polymorphism; uPDI, unhealthy plant-based diet index.
