# Supplementary material for: Metabolomic Signatures of Healthy Dietary Patterns Associated With Lower Risk of Age-Related Macular Degeneration
Source: Invest Ophthalmol Vis Sci. 2026 Nov 4;67(13):11. doi: 10.1167/iovs.67.13.11 (PMC13743827; doi:10.1167/iovs.67.13.11)
Supplement: Supplementary file 2 [file iovs-67-13-11_s002.docx]

**Supplementary Methods**

**1. Dietary pattern scoring**

Dietary intake was assessed using a validated semi-quantitative food frequency questionnaire (FFQ). Each of the eight dietary pattern scores was computed using previously published scoring algorithms, as detailed below.

**Alternative Mediterranean Diet (AMED):** The total AMED score ranged from 1 to 9 and included nine components: vegetables, fruits, whole grains, nuts, legumes, fish and seafood, the ratio of monounsaturated to saturated fat (MUFA:SFA), red and processed meat, and alcohol consumption[23]. Participants received 1 point for intakes above the median for vegetables, fruits, nuts, whole grains, legumes, fish, and MUFA:SFA ratio; otherwise, 0 points. For red and processed meat, consumption below the median was assigned 1 point; otherwise, 0 points. Alcohol consumption was scored as follows: women received 1 point if daily intake was 5–15 g, and men received 1 point if daily intake was 10–25 g; otherwise, 0 points.

**Alternative Healthy Eating Index 2010 (AHEI-2010):** It consists of 11 components, with a total score ranging from 11 to 110: vegetables, fruits, whole grains, sugar-sweetened beverages and fruit juices, nuts and legumes, red and processed meat, trans fat, long-chain n-3 fatty acids, polyunsaturated fatty acids, sodium, and alcohol[24, 25]. Except for trans-fat, long-chain fatty acids, sodium, and alcohol, other food groups adopted serving/d as unit. Each of the 11 components was assigned a minimum score of 1 and a maximum score of 10. For sodium, participants were divided into 11 equal groups based on the distribution of reported intake and assigned scores from 0 to 10, with higher scores indicating lower sodium consumption.

**Dietary Approaches to Stop Hypertension (DASH):** The DASH score comprises 8 components, with a total range of 8 to 40[26]. For fruits, vegetables, nuts and legumes, low-fat dairy products, and whole grains, scores were assigned based on quintile ranking, with the lowest quintile receiving 1 point and the highest quintile receiving 5 points. For sodium, red and processed meats, and sweetened beverages, lower intake was considered favorable: the lowest quintile received 5 points, and the highest quintile received 1 point. The total DASH score was calculated as the sum of the eight food group scores, ranging from 8 (lowest adherence) to 40 (highest adherence).

**Plant-based Diet (PDIs):** PDIs include 18 components and range from 18 to 90[27]. Eighteen food groups were included: 7 healthy plant foods (whole grains, fruits, vegetables, nuts, legumes, vegetable oils, and tea/coffee); 5 unhealthy plant foods (fruit juices, refined grains, potatoes, sugar-sweetened beverages, and sweets/desserts); and 6 animal foods (animal fat, dairy, eggs, fish/seafood, meat, and other animal-based foods). Using an approach similar to that applied for the DASH score, all food components were categorized into quintiles, with each quintile assigned a score from 1 to 5.

**Empirical Dietary Inflammatory Pattern (EDIP):** The EDIP was derived from 39 predefined food groups using reduced-rank regression, followed by stepwise linear regression to identify a dietary pattern most predictive of three plasma inflammatory biomarkers: interleukin-6, C-reactive protein, and tumor necrosis factor-α receptor 2. The final EDIP score was calculated from 18 weighted food groups, with higher scores indicating greater inflammatory potential[28].

**Empirical Dietary Index for Hyperinsulinemia (EDIH):** EDIH was developed and validated to capture the overall insulinemic potential of the whole diet[29]. It was derived from 39 predefined food groups assessed by FFQs, using stepwise regression models to identify a dietary pattern most predictive of fasting plasma C-peptide—a surrogate marker of insulin secretion. The EDIH score was then calculated as a weighted sum of 18 food groups selected.

**2. Ascertainment of age-related macular degeneration**

AMD was graded from color fundus photographs as described in the main text[31, 32]. The detailed grading criteria applied were as follows. The most recent AREDS 2 definitions have been adopted for the study, namely that the standard disc diameter equals 1800 mm (rather than 1500 mm), which affects the size of the Early Treatment Diabetic Retinopathy Study grid and of the standard drusen circles, and that geographic atrophy is present if the lesion has a diameter of 433 mm or more (AREDS circle I-2) and has at least 2 of the following features: absence of retinal pigment epithelium (RPE) pigment, circular shape, or sharp margins (foveal involvement not a requirement). With these criteria, we established the following groups: control (AREDS stage 1), presence of drusen maximum size less than circle C0 and total area less than C1; early AMD (AREDS stage 2), drusen maximum size C0 or more but less than C1 or presence of AMD characteristic pigment abnormalities in the inner or central subfields; intermediate AMD (AREDS stage 3), presence of drusen maximum size of C1 or more or of drusen maximum size of C0 or more if the total area occupied was more than I-2 for soft indistinct drusen and more than 02 for soft distinct drusen; and Late AMD (AREDS stage 4), presence of geographic atrophy according to the criteria described above or evidence of neovascular AMD.
